# Mitophagy and Parkinson's disease: The PINK1–parkin link^☆^

**DOI:** 10.1016/j.bbamcr.2010.08.007

**Published:** 2011-04

**Authors:** Emma Deas, Nicholas W. Wood, Hélène Plun-Favreau

**Affiliations:** Department of Molecular Neuroscience, UCL Institute of Neurology, Queen Square, London WC1N 3BG, UK

**Keywords:** Mitochondria, Mitophagy, Neurodegeneration, Parkinson's disease, Parkin, PINK1

## Abstract

The study of rare, inherited mutations underlying familial forms of Parkinson's disease has provided insight into the molecular mechanisms of disease pathogenesis. Mutations in these genes have been functionally linked to several key molecular pathways implicated in other neurodegenerative disorders, including mitochondrial dysfunction, protein accumulation and the autophagic-lysosomal pathway. In particular, the mitochondrial kinase PINK1 and the cytosolic E3 ubiquitin ligase parkin act in a common pathway to regulate mitochondrial function. In this review we discuss the recent evidence suggesting that the PINK1/parkin pathway also plays a critical role in the autophagic removal of damaged mitochondria–mitophagy. This article is part of a Special Issue entitled Mitochondria: the deadly organelle.

## Research Highlights

► Upon mitochondrial membrane depolarisation, full-length PINK1 accumulates at the outer mitochondrial membrane and NIX translocates to the mitochondria. ► NIX and full-length PINK1 recruit parkin to the mitochondria, which leads to parkin-dependent ubiquitination of VDAC. ► Ubiquitination of VDAC recruits p62 while NIX recruits GABARAP to the mitochondria. ► NIX binds to LC3, which additionally binds p62. ► The combined effects of PINK1, parkin, NIX, VDAC, GABARAP, p62 and LC3 cause depolarised mitochondria to be removed via mitochondrial autophagy – mitophagy.

## 1 Introduction

Parkinson's disease (PD) is the most prominent, progressive movement disorder and the second most common neurodegenerative disease affecting aging populations. The characteristic symptoms of PD include postural instability, rigidity, resting tremor and bradykinesia. A significant proportion of individuals suffering from this disorder appear to have no known genetic cause and are typically referred to as sporadic or idiopathic patients. In contrast to this, 5–10% of individuals with disease are classed as familial patients because they have been shown to carry heritable, disease-associated mutations in a series of genes referred to as the *PARK* genes [1]. To date, six *PARK* genes have been identified which encode: α-synuclein (*PARK*1/4), parkin (*PARK*2), PINK1 (PTEN-Induced Kinase 1—*PARK*6), DJ-1 (*PARK*7), LRRK2 (*PARK*8) and ATP13A2 (*PARK*9). Whilst both LRRK2 and PINK1 are protein kinases, the functions of the remaining genes are diverse and in some cases, still partially unknown. However, mutations in both *LRRK2* and *α-synuclein* result in autosomal dominant inherited disease whilst mutations in *DJ-1*, *parkin*, *PINK1* and *ATP13A2* all give rise to autosomal recessive disease.

Pathologically, PD is characterised by the degeneration of dopaminergic neurons in the substantia nigra accompanied with the presence and accumulation of proteinaceous aggregates, referred to as Lewy Bodies (LB) or Lewy Neurites (LN), in the remaining neurons of affected individuals [2]. Neuropathological analysis has confirmed that α-synuclein positive aggregates are a key component of LB and LN in PD patients and these aggregates are frequently found in other neurodegenerative disorders [2], [3].

A common emerging theme in PD research has been mitochondrial dysfunction and its involvement with disease. Several lines of evidence implicate mitochondria in PD including reduced complex I activity in PD patients [4], reduced mitochondrial membrane potential (ΔΨm) accompanied with increased ROS production in PD cell models [5], [6], alterations in mitochondrial fission–fusion events [7], [8], defects in mitochondrial trafficking [9], [10] and the striking observation that all of the PD related proteins are either mitochondrially located or can associate with mitochondria [11], [12], [13], [14], [15]. Recently, a series of studies involving the PD proteins parkin (an E3 ubiquitin ligase) and PINK1 (a serine/threonine kinase with a mitochondrial targeting sequence), mutations in which are the most common cause of recessive PD [10], [16] have highlighted a potential role for both proteins in the clearance of mitochondria from cells via autophagy—a process known as mitophagy [17]. This observation is intriguing because defects in autophagy/mitophagy have been shown to recapitulate a series of reported PD features namely: impaired motor coordination, tremor and the accumulation of protein aggregates/inclusion bodies in residual neurons [18], [19]. In this article, we review the process of mitophagy and its involvement in neurodegeneration with a particular emphasis on PD.

## 2 A brief word on autophagy

The autophagy-lysosome pathway (ALP) and the ubiquitin-proteasome system (UPS) are the two most important mechanisms that normally remove damaged proteins. Dysregulation of these systems to degrade misfolded and aggregated proteins are being increasingly recognised as playing a pivotal role in the pathogenesis of many neurodegenerative disorders [20].

Autophagy is the process by which unwanted, excess or damaged cytosolic components are ‘self degraded’ by the cell through lysosomal digestion. This catabolic process is a tightly regulated method of maintaining the optimal balance of protein synthesis, degradation and recycling of cellular resources. Autophagy plays a critical role during embryonic and postnatal developmental processes with defects in autophagy frequently displaying aberrant development of the central nervous system (CNS) [21]. Autophagy additionally performs a housekeeping role by eliminating damaged or dysfunctional proteins and/or organelles and has also been implicated in the defence against intracellular pathogen invasion [22], [23], [24]. Whilst autophagy is an ongoing process, which does not require any stress or stimulus for its induction, autophagy can also be induced by a number of conditions including starvation/nutrient deprivation, reactive oxygen species (ROS) production, oxidative stress and pharmacological insult [17], [25], [26]. During nutrient starvation, autophagy facilitates the breakdown of excess proteins or organelles to their component parts and subsequently recycles them to meet the energy requirements of the cell. Three types of autophagy have been defined: macroautophagy, microautophagy and chaperone-mediated autophagy (CMA) [24]. Macroautophagy and microautophagy can recycle cellular components, including whole organelles, via non-selective (housekeeping) or selective (specific targeted degradation) mechanisms [27]. When macroautophagy is employed, unwanted cytosolic material is engulfed and delivered to the lysosome via a double membrane bound vesicle called the autophagosome or autophagic vacuole (AV) [27]. During microautophagy, however, cytosolic components are taken up directly by the lysosome through invagination of the lysosome membrane. In contrast, CMA is a purely selective form of autophagy and can only facilitate the removal of a specific subset of cytosolic proteins containing the signature motif KFERQ [28]. These proteins form a complex with chaperone proteins e.g. Hsc70 in the cytoplasm and are directly transported across the lysosomal membrane. Whilst all three mechanisms are important for cellular function in mammals, dysregulation of macroautophagy has been linked to a series of human diseases. These include numerous forms of cancer and many forms of neurodegeneration [27]. Notably, when no specific reference to the type of autophagy is given in the text, authors refer to macroautophagy as autophagy.

## 3 Macroautophagy and mitophagy

The presence of mitochondria within autophagosome structures was initially observed in mammalian cells by scanning electron microscopy in 1957 [29]. Whilst the notion that mitochondria can be degraded by autophagy is widely accepted, the fact that the process can be selective has only recently come to light and the mechanisms by which this occurs are not yet fully understood.

Over the past decade, our understanding of the molecular mechanisms underlying and regulating macroautophagy has increased dramatically. Studies using the yeast strain *Saccharomyces cerevisiae* (*S. cerevisiae*) provided our first insights into the process and a series of mutation screens have identified 33 AuTophaGy (ATG) genes to date [27], [30]. The identification of the *ATG* genes in yeast has been instrumental in identifying mammalian *ATG* homologues through sequence comparison [31]. In addition, these studies highlighted differences between the autophagic processes and machinery in yeast and mammals such as the existence of an autophagy-like process, exclusively found in *S. cerevisiae*, called the cytoplasm to vacuole targeting (Cvt) pathway (a biosynthetic process which sequesters specific hydrolases into vesicles for subsequent vacuole fusion) and the observation that only a few orthologues of the yeast ATG genes are fully conserved in humans.

The term “mitophagy”, coined by Lemasters in 2005 [17], is defined as “the specific and selective, targeted removal of excess or damaged mitochondria from the cell via autophagy”. Since the discovery of this process, mitophagy is thought to play a role in maintaining a healthy mitochondrial population via removal of dysfunctional/depolarised mitochondria and/or mitochondria producing the highest levels of damaging ROS [32]. In the following section, we summarise what is currently known about mitophagy in yeast and humans and introduce the reader to some of the methods used to study the process.

### 3.1 Mitophagy in yeast

Studies in *S. cerevisiae* provided the first genetic evidence that mitochondrial degradation by autophagy could be a selective process [33]. A number of the *ATG* genes have been reported to play a role in mitophagy in yeast however, these genes are not exclusively involved in mitophagy with many playing additionally roles in the macroautophagy process in general and/or in the Cvt pathway (listed in Table 1). To date, however, loss-of-function screens in yeast have identified five genes involved in mitophagy: *UTH1*, *YmeI*, *AUP1*, *mdm38*/*Mkh1* and *ATG32*.

**Table 1:** Yeast ATG and mitophagy gene involvement in autophagy pathways and identified human homologues.

Yeast gene	Involved in macroautophagy and/or Cvt?	Involved in mitophagy?	Human homologue
ATG 1	√	√	ULK-1, ULK-2
ATG 2	√	√	ATG 2A, ATG 2B
ATG 3	√	√	ATG 3, ATG 3P
ATG 4	√	√	ATG 4A, ATG 4B, ATG 4C, ATG 4D
ATG 5	√	√	ATG 5
ATG 6	√	√	Beclin1
ATG 7	√	√	ATG 7
ATG 8	√	√	LC3B
ATG 9	√	√	ATG 9A, ATG 9B
ATG 10	√	√	ATG 10
ATG 11	√	√	–
ATG 12	√	√	ATG 12, ATG 12P
ATG 13	√	√	–
ATG 14	√	√	ATG 14L
ATG 15	√	√	–
ATG 16	√	√	ATG 16L1, ATG 16L2
ATG 17	√	×	–
ATG 18	√	√	–
ATG 19	√	×	–
ATG 20	√	√	–
ATG 21	√	√	–
ATG 22	×	×	–
ATG 23	√	√	–
ATG 24	√	√	–
ATG 25	√	×	–
ATG 26	×	×	–
ATG 27	√	√	–
ATG 28	√	×	–
ATG 29	√	√	–
ATG 30	√	×	–
ATG 31	√	√	–
ATG 32	×	√	–
ATG 33	×	√	–
UTH1	×	√	–
YmeI	×	√	–
AUP1	×	√	–
Mdm38	×	√	–

Loss-of-function studies in yeast have identified 33 autophagy (ATG) genes and four mitophagy specific genes. Many of the ATG genes perform multiple functions and participate in more than one autophagy pathway. A tick indicates the involvement of the gene whilst a cross shows that loss of this gene has no impact on the autophagy pathway in question. The identified human homologues to these genes are listed.

UTH1 is an outer mitochondrial membrane protein, which in co-ordination with ATG proteins, signals for mitochondria to be selectively removed [34]. In addition, loss of UTH1 function eliminated selective mitophagy indicating that the protein is critically involved in the process [33]. *YmeI* encodes a putative ATPase which, when deleted, induces mitophagy [35]. However, additional studies are required to determine whether mitophagy is initiated as a direct consequence of YME1 protein loss or whether compensation mechanisms inadvertently induce the process. Notably, both UTH1 and YmeI are also reported to play a role in mitochondrial biogenesis but it is currently unclear how these proteins function in this process [36], [37]. *AUP1* (Ancient ubiquitous protein 1) encodes a mitochondrial phosphatase, which was shown to be essential for mitochondrial elimination in non-dividing cells during so called efficient stationary phase mitophagy [38]. AUP1 regulates mitophagy by altering the phosphorylation status of the retrograde signalling pathway (RTG) transcription factor RTG3 [39]. The RTG is a signalling pathway responsible for monitoring mitochondrial function and for inducing nuclear responses during times of mitochondrial stress. Mdm38/Mkh1, a mitochondrial inner membrane protein, is an essential component of the K^+^/H^+^ exchange system. In the absence of mdm38, mitochondria become swollen, lose their ΔΨm and fragment before being removed from the cells via microautophagy. Strikingly, treatment of mdm38 deficient cells with an inhibitor of the K^+^/H^+^ exchanger prevents this form of mitophagy indicating that in yeast, the K^+^/H^+^ exchanger may play an important role in mitochondrial turnover. The most recent yeast mitophagy specific protein to be identified is ATG32 [40], [41]. ATG32 localises to mitochondria and is described as a mitochondrially anchored receptor. Upon induction of mitophagy, ATG32 binds to ATG11, an adapter protein known to be required for selective autophagy. ATG32 has therefore been proposed to function as a mitophagy specific receptor, which regulates the selective removal of mitochondria [41].

### 3.2 Mitophagy in humans

Subsequent studies in mammalian systems have revealed a series of homologues of the yeast *ATG* genes and subsequent molecular studies have reported over 150 additional genes involved in human autophagy. A list of these genes and relevant publications can be accessed via the HADb—Human Autophagy Database (http://www.autophagy.lu/index.test.html). However, to date no homologues of the mitophagy specific genes in yeast (described in 3.1.) have been identified and, through loss of function studies, only two proteins have been reported to directly affect mitochondrial autophagy in mammalian cells: BniP3 (Bcl-2 and adenovirus E1B 19 kDa-interacting protein 3) and NIX/Bnip3L (Bnip3-like protein X).

**Fig. 1:**
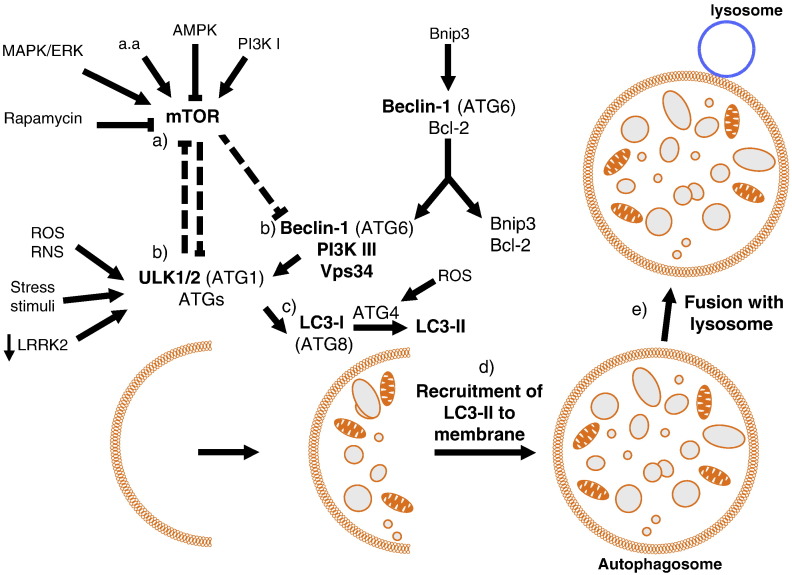
A simplified schematic of macroautophagy in mammals. (a) Activation of autophagy via ULK1/2 is induced by inhibition of the TOR pathway. (b) Autophagosome formation is regulated by the ULK1/2–ATG complex and the PI3kinase complex which includes Beclin1and Vps34. (c) During elongation and closure of the autophagosome membrane, cytosolic LC3-I is cleaved by ATG4 to produce lipidated LC3-II. (d) LC3-II is recruited to the autophagosome membrane. (e) The complete autophagosome fuses with the lysosome to enable content digestion.

**Fig. 2:**
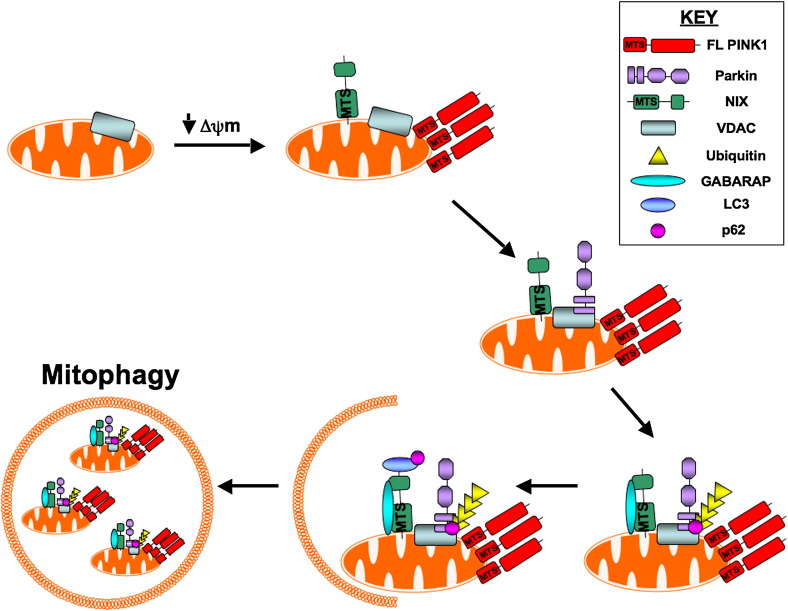
PINK1 and parkin in mitophagy—proposed model. Upon mitochondrial membrane depolarisation, full-length PINK1 accumulates at the outer mitochondrial membrane and NIX translocates to the mitochondria. NIX and full-length PINK1 recruit parkin to the mitochondria, which leads to parkin-dependent ubiquitination of VDAC. Ubiquitination of VDAC recruits p62 while NIX recruits GABARAP to the mitochondria. NIX binds LC3, which additionally binds p62 and depolarised mitochondria are removed via autophagy.

BNIP3 and NIX are both pro-apoptotic members of the Bcl-2 family of proteins. They each contain a BH3 only domain and a mitochondrial targeting sequence, which localises them to mitochondria [42] (and this issue of BBA). BNIP3 has been linked to hypoxia-induced mitophagy in cardiomyocytes. In these studies, increased Bnip3 expression correlated directly with a decrease in mitochondrial DNA copy number which was accredited to mitochondrial removal via autophagy [43]. In addition, Bnip3 interacts with the anti-apoptotic protein Bcl-2. Interaction of Bnip3 with Bcl-2 abrogates the ability of Bcl-2 to remain bound to the autophagy protein Beclin-1 (ATG6), which promotes autophagy once released [44] (Fig. 1). In contrast, examination of *NIX* deficient mice revealed that loss of NIX function impairs normal reticulocyte development through defects in mitochondrial clearance and mitochondria predominantly associate with outer autophagosomal membranes [45]. This observation led to the hypothesis that NIX is required for mitochondrial incorporation into autophagosomes during reticulocyte development and that a lack of mitophagy is deleterious to cells. Importantly, subsequent work has demonstrated that NIX interacts with the autophagy specific ubiquitin like proteins LC3 and GABARAP, which are required for autophagosome formation [46], [47]. The interaction between NIX and LC3 is strengthened under conditions of mitochondrial stress or in the presence of pharmacological agents which block autophagy, whilst GABARAP was shown to be recruited to depolarised mitochondria in a NIX dependent manner (Fig. 2) [47]. Novak et al. therefore conclude that NIX is a selective autophagy receptor, which mediates the removal of damaged mitochondria. NIX expression, which can be induced by cell stress, growth or differentiation, promotes outer mitochondrial membrane permeabilisation and allows the release of cytochrome *c* to induce apoptosis. Importantly, normal reticulocyte development could be restored in *NIX* deficient cells by treatment with pharmacological agents, which depolarise mitochondria. This observation therefore suggests that NIX may initiate mitophagy via mitochondrial depolarisation. Notably, NIX expression was upregulated in a PD model system where neuronal PC12 cells were exposed to 6-hydroxydopamine (a neurotoxin which specifically kills dopaminergic and noradrenergic neurons) [48]. In this system, however, NIX interacts with POSH (plenty of SH3 domains), which is known to play a role in activation of the JNK/c-Jun apoptotic pathway, and drives neuronal death via c-Jun activation. In addition, expression of a dominant negative NIX mutant, which can no longer target to mitochondria, protects against neuronal death in this system. These results suggest that loss of NIX function could be protective in neurons subjected to stress. However, whether dysregulated mitophagy plays a role in neuronal death has yet to be investigated in this system. Recently, NIX was also reported to play a role in the translocation of the PD associated protein parkin to mitochondria (see Section 6.1 and Fig. 2) and for induction of autophagy via ROS stimulation [49]. Through a series of recent studies, parkin may represent a new mitophagy specific protein in mammals (addressed in Section 6.1).

### 3.3 Assessing mitophagy in mammalian systems

Studies investigating mitochondrial turnover/degradation by autophagy have typically been performed in cell types/tissues that naturally eliminate mitochondria during their maturation or development e.g. lens, erythrocytes and sperm [37]. In cell types where this does not occur, cell stressors have been utilised to induce autophagy and mitochondrial turnover is subsequently assessed. The most common method to induce autophagy is through inhibition of the target of rapamycin (TOR) kinase pathway. Inhibition of TOR via starvation/nutrient deprivation or rapamycin treatment activates the ULK1 kinase and induces autophagosome production (Fig. 1) [50].

Initial studies by Lemasters et al. implicated opening of the mitochondrial permeability transition pore (PTP) to mitochondrial autophagy [51], [52]. However, subsequent conflicting reports have emerged and at present there is still debate over the involvement of PTP in mitophagy initiation [53]. A common mitochondrial feature, which appears to accompany mitophagy in human cells, is a reduction or loss of ΔΨm. Therefore, pharmacological treatments, which induce mitochondrial membrane depolarisation e.g. uncoupling reagents such as FCCP and CCCP, are also frequently used to assess mitophagy. In addition, mitophagy can also be induced by photo damage, ROS (e.g. H_2_O_2_) production, the MEK/ERK kinase signalling pathway and increased or induced mitochondrial fission [32], [53], [54]. Irrespective of the stimulus, the assays used to assess mitophagy frequently utilise markers of autophagosomes to monitor the process. Autophagy can be assessed by electron microscopy, immunofluorescence and biochemical approaches. A comprehensive description of these methods, along with their pros and cons was published a few years ago and we would subsequently recommend it to interested parties [55]. However, for the purpose of this review, we briefly introduce two of the methods used to assess mitophagy in recent PD studies below.

Microtubule-associated protein light chain 3 (MAP-LC3, LC3) is the mammalian homologue of ATG8. Three forms of LC3 exist: the full-length newly synthesised protein (LC3), a soluble form generated by cleavage at the C-terminus by ATG4B (LC3-I) and a lipidation form (LC3-II) [25], [56]. LC3-I, which resides in the cytoplasm, is converted to LC3-II through two-ubiquitin like modifications, which covalently link the protein to phosphatidylethanolamine (PE) [24]. Importantly, LC3-II then localises to autophagosomal membranes and thus the levels of LC3-II in a cell are frequently used to demonstrate induction of autophagy [57]. Fluorescently labelled LC-3II (GFP-LC3-II) is one of the most common methods for assessing autophagy via microscopy and live imaging experiments. In addition, the presence of fluorescent mitochondrial markers (e.g. mitotracker, DsRed-mito) within a GFP-LC3-II labelled autophagosome is frequently used to demonstrate mitophagy in mammalian systems [58].

Sequestosome 1 (P62/SQSTM-1) is a multi-functional adaptor protein, which interacts with ubiquitinated proteins and is thought to be involved in the delivery of ubiquitinated proteins/protein aggregates to both the ubiquitin proteosome system (UPS) or autophagosome for degradation [59]. P62 interacts with LC3-II, becomes incorporated into the autophagosome and subsequently degraded [60]. Impaired autophagic activity leads to an accumulation of p62 in cells [55]. Consequently, the levels of p62, assessed by Western blot or immunofluorescence, are frequently used as a marker for impaired/defective autophagy. Through studies in neurodegenerative models, P62 is thought to function in autophagy as a selective means to sequester ubiquitinated protein aggregates into autophagosomes and has been shown to accumulate in the LB/LN of PD patient neurons [61], [62].

## 4 Autophagy and mitophagy in neurons

To date, the majority of investigations to unravel the mechanisms underlying mitophagy, and hopefully elucidate its role in disease pathogenesis, have been performed in mitotic cells. These studies have been instrumental in demonstrating that autophagy and mitophagy are differentially regulated in different tissues/cell types and therefore the study of these processes in post-mitotic long-lived cells such as neurons is critical. Assessment of autophagy in the CNS, using *ATG5* and *ATG7* knockout mice, revealed that an autophagic deficit results in neuronal degeneration and the accumulation of ubiquitin labelled intracytoplasmic inclusion bodies in remaining neurons [18], [19]. These studies demonstrate the critical role autophagy plays in maintaining neuronal health (for a detailed review see [60]) but also highlight the fact that not all neurons show the same level of susceptibility to impaired autophagy. There are three potential explanations for the differential susceptibility of neuronal populations. One is that different types of neurons employ different compensatory mechanisms in response to impaired autophagy. Alternatively the local environment of the neurons could affect how the neurons respond to stress or toxic insult. However, the observed differences could also indicate that different types of neurons have differential rates of basal autophagy and therefore neurons which are subjected to higher levels of stress and subsequently require higher levels of autophagy, are more at risk of damage/death over time. Critically, autophagy is known to decrease with age [63]. Therefore, if the latter explanation is true, this could additionally explain why subsets of neurons are also at risk of irreparable damage/death during the human lifespan. Investigations to identify the reason behind selective neuronal degeneration in the *ATG* knockout mouse models could provide a great deal of insight into the selective loss of neuronal populations in human disease e.g. dopaminergic neurons in PD. However, one interesting study in mice showed that male neurons are more susceptible to autophagic death than female neurons [64], which implies that gender specific differences could also play a role in differential regulation of autophagic processes. As PD predominantly affects male members of the population, it would be interesting to assess if the observations in mice are recapitulated in humans. If this is the case then studies using healthy mouse neurons could potentially aid our understanding of the gender specific susceptibilities and aid in the development of therapeutics.

Notably, autophagosomes are rarely observed in healthy neurons. At present, the reasons behind this are unknown but several hypotheses have been put forward to explain this observation. In the first scenario it is assumed that a lack of autophagosomes indicates that neurons have high basal autophagic rates compared to other cell types and autophagic material is rapidly cleared from the cell [60]. However, the absence of autophagosomal structures could also indicate that neurons utilise an alternative, as yet unidentified, autophagic pathway. Studies have shown that, in neurons, a starvation stimulus does not induce LC3-II labelled autophagosomes and under normal conditions LC3 exists predominantly in its soluble LC3-I form [65]. LC3-I is known to bind to microtubule associated protein 1B (MAP1B), a protein expressed at high levels in neuronal cells. The production of LC3 positive autophagosomes can be reduced through MAP1B expression and hence the enriched MAP1B environment of neurons may also explain the lack autophagosomes [65], [66]. In addition, a key regulator of autophagy in neurons is insulin [66]. Loss of insulin signalling directly impacts on the TOR pathway and results in neuronal autophagy. The lack of autophagosome formation upon a starvation stimulus is thought to be due to compensatory mechanisms of nutrient supply from surrounding astrocytes and peripheral organs.

At present, little is known about the regulation or dysfunction of mitophagy in neurons. Given their high dependence on mitochondria however, one would assume that an inability to remove dysfunctional/damaged mitochondria from the cell would have severe and detrimental consequences over time. In several models of PD, enlarged or swollen mitochondria have been reported [7], [8], [67], [68], [69]. This observation potentially highlights a significant role for mitophagy in PD pathogenesis. In addition, a study by Liang et al. reported that in healthy mice, substantia nigra DA neurons have a reduced mitochondrial mass compared to other neurons in the substantia nigra [70]. We are not aware of alterations in mitochondrial mass in human DA neurons, but if these neurons do have smaller mitochondrial populations, it may explain why these cells are particularly sensitive to mitochondrial dysfunction. A comparative analysis of mitochondrial mass in DA neurons, and additional neurons degraded in PD would therefore be of interest. However, key studies investigating mitophagy in neurons under healthy physiological conditions are lacking and as a result, it is unclear where the problem initiates at the molecular level. Importantly, a critical balance between mitophagy and mitochondrial biogenesis must be maintained to meet the energy requirements of the cell. Failure to maintain an optimal healthy mitochondrial population, through defects in either mitophagy or biogenesis could explain why specific tissues like the brain, which critically depend on mitochondrial function, are most vulnerable to dysfunction in aging organisms.

## 5 Autophagy and neurodegenerative diseases

Protein aggregation, mitochondrial impairment and oxidative stress are common to a number of neurodegenerative diseases. Specifically protein aggregation presents one of the common features in numerous neurodegenerative disorders including the α-synuclein containing LB/LN in PD, the beta-amyloid plaques in Alzheimer's disease (AD) and the mutant huntingtin cytoplasmic inclusions in Huntington disease (HD).

Defective autophagy was initially implicated in neurodegenerative disease because increased levels of autophagic vacuoles were observed in AD, HD and PD patient brain tissues as well as in animal models of each disease (reviewed in [71]). Moreover it has been shown that knockdown of *ATG* genes resulted in neurodegeneration and the presence of cytoplasmic inclusions (discussed in Section 4) [18], [19].

Both α-synuclein [72], [73], [74] and superoxide dismutase (mutations in the *SOD1* gene encoding superoxide dismutase are associated with familial amyotrophic lateral sclerosis or ALS) [75] are eliminated by autophagy. In addition, the PD related kinase LRRK2 has recently been shown to regulate autophagy and localise to autophagic vacuoles [76], [77]. Alegre-Abarrategui et al. have demonstrated that loss of LRRK2 resulted in an increase in autophagic activity in human cells (Fig. 1). Importantly, expression of LRRK2 carrying either the G2019S or R1441C PD related mutation, resulted in a significant accumulation of autophagic vacuoles within cells [77]. Dysregulation of the ALP was also reported in the knockout LRRK2 mouse model where a significant amount of aggregated α-synuclein and ubiquitinated proteins accumulated in the kidneys of these animals in an age-dependent manner [76]. In contrast to the findings of Alegre-Abarrategui et al., however, these autophagy-lysosomal defects were not observed in the R1441C knock in mouse model and hence the impact of LRRK2 PD related mutations on ALP requires further investigation [76]. As mentioned previously, TOR is a pivotal player in autophagy [78] and notably induction of TOR-dependant autophagy via rapamycin leads to a decrease of mutant huntingtin aggregates and prevents neurodegeneration in a mouse model of HD [79]. Furthermore trehalose, a TOR-independent autophagy enhancer was shown to accelerate the clearance of mutant huntingtin and α-synuclein [80]. The authors have further shown that trehalose and rapamycin together exert an additive effect on the degradation of these aggregate-prone proteins due to increased autophagic activity [80]. If defective autophagy plays a crucial role in neurodegeneration in general, its implication in PD in particular is increasingly recognised. Evidence for this comes from recent studies showing that administration of l-DOPA, a precursor to the neurotransmitter dopamine clinically used to treat PD patients, in a mouse model of parkinsonism led to dopamine D1 receptor-mediated activation of the TOR pathway [81]. In addition, inhibition of TOR activation via rapamycin treatment, protected against neuronal death in toxin cell and animal models of PD [82], [83]. Two subsequent studies in *Drosophila* have strengthened the link between PD-associated proteins and TOR-dependent signalling. Effectively Imai et al. have reported 4E-BP, a member of the TOR pathway, as a potential substrate of LRRK2 in *Drosophila* [84]. However whether 4E-BP is a direct authentic substrate of LRRK2 remains unclear [85]. Further experiments in *Drosophila* showed that 4E-BP over-expression could rescue *PINK1* and *parkin* knockout phenotypes [86]. All together these studies suggest a link between some of the PD-associated genes and the TOR pathway in *Drosophila*. Further studies will be required to confirm this possible functional interaction between PD-associated genes and TOR in mammals. Finally TOR-dependant signalling has also been implicated in other neurodegenerative diseases such as AD and ALS [87], [88].

The lysosomal system is essential for completion of autophagy-initiated protein and organelle degradation and there is increasing evidence that lysosomal dysfunction is another important process in a variety of neurodegenerative diseases, including AD, PD, HD, and ALS [89]. One of the examples for neurodegeneration caused by deficiency of enzymes in lysosomes is lysosome storage disorder, which is an inherited metabolic disease, characterised by accumulation of various toxic materials in neurons as a result of the lysosomal enzyme deficiencies [90], [91]. It has been shown that wild-type α-synuclein, but not the mutant α-synuclein, is selectively translocated into lysosomes for degradation by the CMA pathway [92]. Moreover Xilouri et al. have shown that expression of mutant α-synuclein and in some cases wild-type α-synuclein induced CMA dysfunction, therefore mediating lysosomal dysfunction and eventually leading to neuronal death [93]. It has also been shown that lysosomal dysfunction accompanies α-synuclein aggregation in a progressive toxic mouse model of PD [94], and that mutations in *ATP13A2*, another PD-associated gene encoding a lysosomal ATPase, lead to defective autophagy and aggregation of α-synuclein in PD [95], [96]. Ultrastructural studies of PINK1-deficient cells often display an increased lysosomal content [97], [98], [99]. Finally mutations in the gene encoding glucocerebrosidase (GBA), the enzyme deficient in the lysosomal storage disorder Gaucher disease have recently been shown to be associated with the development of PD [100]. All together these observations support the hypothesis that lysosomal malfunction is another important cause of neurodegenerative diseases, in particular PD.

In summary the fact that decreased autophagic activity (as demonstrated by accumulated autophagosomes) was found in degenerating neurons of human brains suffering from AD, PD and HD supports the concept that autophagy may act as a protective aggregate clearance pathway. Thus as a result of defective autophagy, protein plaques and aggregates would accumulate in neurons, resulting in neurodegeneration (reviewed in [101]).

## 6 Mitophagy and neurodegenerative diseases

Aside from the degradation of cytosolic aggregates, the autophagic pathway is also involved in the turnover of entire organelles such as mitochondria. Mitophagy is, to date, the only identified mechanism by which mitochondria are recycled. Alterations in mitochondrial function and dynamics as well as in the mitophagic pathway are increasingly recognised as being implicated in a number of neurodegenerative diseases [102], [103], [104]. For example mitochondria have been shown to be key targets of increased autophagic degradation in AD and PD [105]. In the following section we focus on recent discoveries highlighting the emergent role of mitophagy in PD pathogenesis.

### 6.1 Mitophagy and Parkinson's disease—PINK1 and Parkin

As mentioned in Section 3.2, the precise molecular mechanisms for mitochondrial recognition by autophagy are just beginning to be elucidated. However, Chu et al. have shown that when autophagy was induced by the parkinsonian neurotoxin MPP+, mitochondrial degradation could be inhibited by knockdown of ATG genes. Furthermore this study implicated that mitophagy occurred independently of the PI3K/Beclin1 pathway but was dependant upon MAPK/ERK signalling instead [106]. The PI3K/Beclin1 kinase complex is known to be involved in regulating autophagosome formation in mammals (Fig. 1). Beclin1 is known to interact with full length PINK1 which drives autophagy in mammalian cells, however, whether PINK1 is involved with the PI3K/Beclin1 complex is currently unknown [107]. Notably PI3K/Beclin1-independent MAPK/ERK-dependent autophagy was also observed in neurite retraction elicited by the G2019S PD mutant of LRRK2 [108]. Interestingly Zhu et al. had already shown that phosphorylated ERK was localised to mitochondria and autophagosomes in Lewy body diseases [109]. Dagda et al. have further shown that mitochondrial localisation of active ERK2 was necessary to increase toxin-induced mitophagy in the dopaminergic-like neuronal cell line SH-SY5Y [54]. Whether ERK promotes diffuse or mitochondria-selective autophagy remains unclear and understanding the mechanisms by which ERK promotes mitophagy as well as identifying mitochondrial specific targets of ERK may offer avenues for future research in PD.

The most recent candidate for ATG5-dependant mitophagy in mammals is parkin [110], which may clear damaged mitochondria by recruiting proteins such as ubiquitin and p62 [111]. Through a series of high-resolution imaging experiments, Narendra et al. first demonstrated that, although the E3 ubiquitin ligase parkin was primarily cytosolic, it was selectively recruited to damaged or depolarised mitochondria in mammalian cells. Moreover the authors demonstrated that after recruitment, parkin was responsible for mediating the uptake of mitochondria by autophagosomes and their subsequent degradation [110], [112]. In this study, parkin recruitment to the mitochondria was shown to be voltage-dependent and independent of ATP levels or pH. All together these findings suggest that parkin-dependant elimination of dysfunctional mitochondria with a decreased mitochondrial membrane potential may be implicated in the pathogenesis of PD [113].

Although prior studies suggest that PINK1 and parkin interact genetically to regulate mitochondrial function [7], [114], [115], [116], the molecular pathway/s used by PINK1 and parkin to regulate mitophagy remained unknown. A number of recent studies have however provided some molecular clues on how PINK1 may regulate parkin-mediated mitophagy and how PD-associated *PINK1* and *parkin* mutations result in defective mitophagy. Dagda et al. have shown that stable knockdown of *PINK1* in SH-SY5Y cells induced mitophagy through effects on oxidative stress and mitochondrial fission whereas overexpression of *PINK1* stabilised mitochondrial networks. They have also shown that parkin further enhanced this protective mitophagic response, suggesting that PINK1 and Parkin may cooperate to maintain mitochondrial homeostasis [117]. Interestingly, PINK1 and parkin have the opposite effect in *Drosophila* (with loss of function inducing mitochondrial fusion and overexpression driving fission) reviewed in [10]. Whilst the roles of PINK1 and parkin in mitophagy have not yet been described in *Drosophila*, future studies to investigate their role/s in the process should prove exceptionally interesting to the field. Vives-Bauza et al. demonstrated that the relocation of parkin to mitochondria was dependent on PINK1 expression and that mutations in either *PINK1* or *parkin* impaired mitochondrial trafficking, especially in the perinuclear region, a sub-cellular area where defective mitochondria could be targeted for autophagy [118]. Furthermore Lin and Kang have demonstrated that PINK1 cleavage was inhibited in damaged mitochondria that had a decreased mitochondrial membrane potential [119]. Narendra et al. have further shown that full length PINK1 accumulates on these dysfunctional mitochondria, leading to the recruitment of parkin at the outer membrane of these damaged mitochondria and eventually their degradation by mitophagy (Fig. 2) [120]. Similarly, a reduction in mitochondrial membrane potential was reported to stabilise PINK1 mitochondrial accumulation. As a result parkin is recruited from the cytosol to the depolarised mitochondria where it becomes active and can initiate mitophagy. Notably, PINK1-dependent mitochondrial localisation has been shown to be necessary to liberate the latent ubiquitin ligase activity of parkin [121]. These combined studies would therefore suggest that accumulation of full-length PINK1 at the mitochondria is required to recruit parkin to the organelle. Geisler et al. have further shown that PINK1 kinase activity and its mitochondrial targeting sequence were necessary to induce translocation of parkin to depolarised mitochondria, which further requires parkin E3 ubiquitin ligase activity. In addition they have shown that the mitochondrial voltage-gating channel VDAC (voltage-dependent anion channel 1) was a target for parkin-mediated Lys27 poly-ubiquitination and p62-mediated autophagy (Fig. 2) [111]. However, a recent conflicting study in primary dermal fibroblasts originating from PD patients demonstrated that mitochondrial accumulation of full length PINK1, whilst sufficient, was not necessary for the stress-induced mitochondrial translocation of parkin. Furthermore the authors showed that parkin mitochondrial translocation was independent of the mitochondrial membrane potential [122]. More recently, NIX, a protein already implicated in mitophagy (see Section 3.2) was reported to be critical for parkin translocation to the mitochondria (Fig. 2). Notably, the C-terminus of NIX, which is required for localisation of NIX to mitochondria, is required for parkin translocation [49]. In addition, a role for parkin in the selective removal of mitochondria harbouring mtDNA mutations has recently been reported [123]. In this study, parkin overexpression was shown to eliminate mitochondria with deleterious mtDNA mutations thereby ensuring the maintenance of a healthy mitochondrial population. Notably, patients heterozygous for *PINK1* have been reported to harbour mtDNA mutations [124]. If PINK1 is involved in parkin translocation to mitochondria, then the deficit in PINK1 in these patients may explain the retention of deleterious mtDNA mutations in PINK1 PD patients.

However, the conflicting views, regarding the mechanism regulating parkin translocation to mitochondria, highlight the need for more experiments to be performed where protein function can be assessed at the endogenous level and in a neuronal setting. Unfortunately with regards to PINK1, the scientific community has so far been limited by the difficulty of detecting the endogenous protein. This is due to the absence of an antibody capable of detecting PINK1 in a specific and reproducible manner but additional problems are incurred due to the short half-life of PINK1 and its cleavage products (approx 30 min) [119]. Full length PINK1, but not its cleaved forms have recently been shown to enhance autophagy through its interaction with Beclin1, a key pro-autophagic protein already implicated in the pathogenesis of AD and HD [107]. Although strengthening the link between PINK1 and autophagy, it is difficult to correlate these findings with the study of Chu et al., which shows that MPP+ induced autophagy is Beclin1 independent [106]. This discrepancy simply highlights the fact that the macroautophagy process, observed in PD, is probably far more complex than previously thought, with various autophagy pathways being implicated in the process.

Importantly the study performed by Geisler et al. provides a missing link between ubiquitin and the two degradation systems, UPS and ALP [111]. In *Drosophila* cells ubiquitination of the profusion factor mitofusin may provide another mechanism by which damaged mitochondria are targeted for degradation by PINK1/parkin-dependent autophagy [125]. Together, these studies suggest that mutations in either *parkin* or *PINK1* may alter mitochondrial turnover, which in turn, may cause the accumulation of defective mitochondria and, ultimately, neurodegeneration in PD (for comprehensive reviews, [101], [126], [127], [128]. Although these studies represent a major step forward towards defining the cellular function of the PINK1/parkin pathway in removal of dysfunctional mitochondrial by mitophagy, many issues remain to be clarified.

## 7 Future perspectives—mitophagy and PD

Over the past few years, the involvement of PD associated genes with autophagy and mitophagy has come to light. Whilst the roles that these proteins play are beginning to be understood, a series of questions remain to be addressed. Predominantly, the mechanisms and conditions under which parkin gets recruited to the mitochondria remain unclear. Effectively, since parkin does not have a predicted mitochondrial targeting sequence (MTS), the current studies implicate PINK1, NIX or environmental stress as possible drivers of parkin to the outer mitochondrial membrane (omm). Subsequent investigations to reveal whether endogenous PINK1 and/or NIX interact with parkin on their way to the mitochondria or exclusively at the mitochondria and whether parkin can still translocate to mitochondria in NIX deficient neurons would help to clarify the situation. Notably, PINK1 was reported to phosphorylate parkin on Thr175, which promoted parkin localisation to mitochondria [14]. In this study, parkin phosphorylation by PINK1 was assessed *in vitro* and at present these data have not been reproduced *in vivo*. Given the recent findings that NIX has been shown to be essential for parkin mitochondrial translocation, it would be interesting to assess whether the interaction between parkin and NIX is dependent on parkin's phosphorylation status or whether NIX perhaps functions as an adaptor to aid parkin phosphorylation. PINK1 kinase activity has been shown to regulate the function of several mitochondrial proteins, such as the TRAP1 chaperone protein and the Omi/HtrA2 protease [129], [130]. It has therefore been proposed that PINK1, HtrA2 and parkin interact to maintain mitochondrial homeostasis [117]. Interestingly, a recent study showed that Omi/HtrA2 could promote autophagy, therefore facilitating the degradation of mutant α-synuclein and truncated huntingtin, proteins associated with PD and HD respectively [131].

One inconsistency raised by the studies on PINK1 and parkin is the observation that damaged mitochondria can stabilise full length PINK1 in the outer mitochondrial membrane. Lin and Kang reported that the integrity of the mitochondrial membrane was crucial for PINK1 cleavage and, under depolarising conditions, full-length PINK1 accumulated on the outer mitochondrial membrane [119]. Whilst the results of Narendra et al. support these findings, the study by Vives-Bauza et al. showed that the levels of both full-length and cleaved PINK1 increased upon mitochondrial depolarisation [118]. Further studies to assess the proteolysis and proteosome-dependent clearance of PINK1 under normal and mitochondrial-depolarising conditions will therefore be required to clarify the issue.

The additional queries of whether mitochondria necessarily need to be depolarised in order to be targeted for PINK1/parkin-dependent mitophagy is a prominent area where additional investigations are needed. Notably in the absence of mitochondrial membrane depolarisation, several manipulations that increase the levels of PINK1 at the omm are sufficient to recruit Parkin to mitochondria and promote mitophagy [120]. Other PD-associated proteins, such as DJ-1, LRRK2 and α-synuclein have been reported to be at least partially located to the mitochondria and to interact with PINK1 and/or parkin in mammalian cells [99], [132], [133], [134], [135]. Whether these proteins interact with the PINK1/parkin mitophagy pathway remains to be clarified.

At present, the major limitation of most of the studies on PINK1/parkin-dependent mitophagy is the reliance on overexpression of *parkin* or *PINK1* in mitotic cells. To clarify many of the issues raised above there is an indispensable need to re-evaluate the selective mitophagy pathway in post-mitotic neurons expressing the endogenous wildtype or mutant parkin and PINK1 proteins. The use of iPS-differentiated neurons from normal individuals and patients with mutations in the PD-associated genes is a very promising tool and the production of knock-in mouse or rat models for these PD genes would be indispensable due to the limited availability of patient material. Notably there is a desperate need for a specific antibody capable of reliably detecting the PINK1 protein at the endogenous level in mitotic and post-mitotic neuronal cell lines, which are better able to recapitulate neurodegenerative disease.

In conclusion, there is increasing evidence that autophagy, and mitophagy with respect to PD, are critically linked to the pathogenic mechanisms underlying neurodegenerative disease. An important question currently being raised, however, is whether autophagy or mitophagy perform a protective role or whether their dysregulation is in fact a common underlying cause of disease initiation. Given the demonstration that activation of macroautophagy, in a mouse model of HD, reduced both the presence of protein aggregates and neuronal loss [79], suggests that modulation of the autophagy pathway in mammals is possible and could be beneficial in slowing down disease pathogenesis. However, the potential to induce cell death via excessive autophagic stimulation is a significant risk and a much greater understanding of how the process is regulated in multiple cell types is crucial before beneficial alterations can be induced via therapeutics. With regards to mitophagy, it is clear that mitochondrial dysfunction is directly linked to neurodegeneration and PD pathogenesis in particular [102], [103]. Neurons critically depend upon mitochondrial respiration for energy and failure to eliminate mitochondria, which have respiration defects and/or generate increased levels of damaging ROS, will impose a significant oxidative burden over time. Our current understanding of the mitophagy process, specifically which factors influence and signal its initiation are still in their infancy. If, through further studies, we can unravel the molecular mechanisms underlying both mitophagy and mitochondrial biogenesis, and develop methods to delicately modulate both processes, it may be possible to enrich neuronal cells with the healthiest mitochondrial population available (as demonstrated by the recent in vitro study by Suen et al. [123]—Section 6.1). However, driving mitophagy in a cell, which already has a severely damaged mitochondrial population, without mitochondrial biogenesis, would most likely eliminate a significant number of the organelles and result in neuronal death. Nevertheless, when we take into account the specific regulation of mitophagy in different cell types, this opens up an attractive avenue for therapeutic intervention where modulation of the process could have the potential to target certain cell populations without inducing detrimental effects on the remaining organs and tissues of the brain/body.
